# Keypoint-Based Robotic Grasp Detection Scheme in Multi-Object Scenes

**DOI:** 10.3390/s21062132

**Published:** 2021-03-18

**Authors:** Tong Li, Fei Wang, Changlei Ru, Yong Jiang, Jinghong Li

**Affiliations:** 1 College of Information Science and Engineering, Northeastern University, Shenyang 110819, China; 1970644@stu.neu.edu.cn (T.L.); 1870652@stu.neu.edu.cn (C.R.); lijinghong@ise.neu.edu.cn (J.L.); 2 Faculty of Robot Science and Engineering, Northeastern University, Shenyang 110169, China; 3 State Key Laboratory of Robotics, Shenyang Institute of Automation, Chinese Academy of Sciences, Shenyang 110016, China; jiangyong@sia.cn

**Keywords:** robot grasping, CNN, keypoint, multi-object scenes, Cornell dataset, VMRD

## Abstract

Robot grasping is an important direction in intelligent robots. However, how to help robots grasp specific objects in multi-object scenes is still a challenging problem. In recent years, due to the powerful feature extraction capabilities of convolutional neural networks (CNN), various algorithms based on convolutional neural networks have been proposed to solve the problem of grasp detection. Different from anchor-based grasp detection algorithms, in this paper, we propose a keypoint-based scheme to solve this problem. We model an object or a grasp as a single point—the center point of its bounding box. The detector uses keypoint estimation to find the center point and regress to all other object attributes such as size, direction, etc. Experimental results demonstrate that the accuracy of this method is 74.3% in the multi-object grasp dataset VMRD, and the performance on the single-object scene Cornell dataset is competitive with the current state-of-the-art grasp detection algorithm. Robot experiments demonstrate that this method can help robots grasp the target in single-object and multi-object scenes with overall success rates of 94% and 87%, respectively.

## 1. Introduction

Grasping is one of the main ways for robots to interact with the real world. Moreover, robotic grasping is often used in industrial and service environments, such as warehouses, homes, etc. In order to better achieve human-machine cooperation under household or industrial scenes, it is important for service robots or industrial robots to be able to grasp specified objects in complex scenes containing multiple objects. This requires three issues to be solved: (1) how to accurately detect the category and grasps of objects in multi-object scenes; (2) how to determine the belonging relationship between the detected objects and the grasps; (3) how to obtain the executable grasp of the specified object. Human can stably and accurately grasp a specific target even in a constantly changing environment, however, it’s still challenging for robots to solve this problem. To grasp an object properly, the robot should first accurately recognize it and determine the grasp type before grasping. Therefore, we propose a new scheme to identify the grasps of a specific target in multi-object scenes.

Previous research works [1,2,3,4,5,6] focused on grasp detection in single object scenes. This method does not need to distinguish the category of the object and directly detects the grasping pose of the object. Moreover, on the Cornell dataset [2], the current state-of-the-art grasping detection algorithm achieves an accuracy of 97.74% on image-wise split and 96.61% on object-wise [6]. However, in practical applications, robots often face multi-object scenes. For this problem, some studies [7,8,9,10,11] have been proposed to solve the grasp detection in multi-object scenes. However, these studies only detect the grasping and did not know the category of the grasped object, that is, what to see and what to grasp. However, in reality, what is needed is to know how to grasp and what to grasp. Moreover, Zeng et al. [12] proposed a “grasp-first-then-recognize” framework. Instead of first recognizing, it directly grasps an object from a bunch of objects, and then judges its category. However, when used in scenes with many targets, the process of detecting one by one limits the efficiency. Recently, Zhang et al. [13] proposed a region-of-interest (ROI) based robot grasping detection algorithm ROI-GD, which has a good performance in detecting the grasps of specific targets in multi-object scenes. However, like most previous methods, ROI-GD uses an anchor-based detection method. This method relies on excessive manual design, enumerating an almost exhaustive list of potential object locations, and processing each location. This is wasteful, inefficient, and requires additional post-processing. Moreover, because the location of anchor is not sufficient, there are inherent shortcomings.

**Figure 1:**
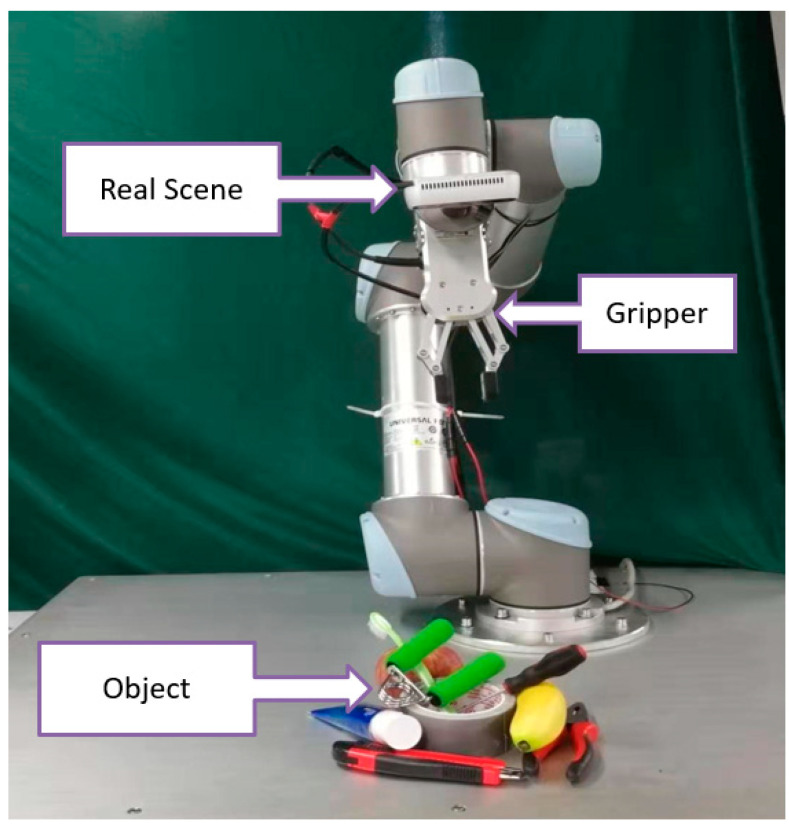
Robotic grasping environment.

For robot grasping detection, a good grasping point determines a good grasping, and only the correct grasping point can get the correct grasping. Therefore, we propose a new robot grasping detection method based on keypoints. We model an object or a grasp as a single point—the center point of its bounding box. The detector uses keypoint estimation to find the center point and regress to all other object attributes such as size, direction, etc. On the Cornell dataset, the accuracy of our scheme in image splitting and object splitting is 97.12% and 95.89%, respectively, which is equivalent to the most advanced grasping detection algorithm. In order to verify the effect of the scheme in multi-object scenarios, we used the VMRD dataset [14] containing multi-object scenarios, which produced an accuracy of 74.3%. As shown in Figure 1, our method is also applied to real robot grasping tasks. UR5 is used as an actuator to complete the grasping task, in which the robot is required to detect the grasping of specified objects, develop a grasping plan, and complete the grasping step by step. In the single-object scene, the grasping success rate was 94%, and in the multi-object scene, the grasping success rate was 87%.

In summary, contributions of this paper include:

- A new scheme of robot grasping detection based on keypoint, which is different from the anchor-based scheme in the previous work, is proposed.
- A new network is proposed that detects objects and grasp at the same time and matches the affiliation between them in multi-object scenes.
- A matching strategy of objects and grasps is proposed, which makes the result more accurate.

The rest of the paper is organized as follows: The related work is discussed in Section 2. Our proposed approach is illustrated in Section 3. Detailed experiments setup is represented in Section 4. We present our results in Section 5, then conclude in Section 6.

## 2. Related Works

In previous works [15,16,17] model-based methods have taken a dominant position in solving the problem of grasping. This method uses the complete 3-D model of the object to model the grasping operation. Considering many constraints, an objective function is established to optimize the stable grasping pose. However, the environment the robot faces is unknown, and it is impossible to obtain a 3-D model of the object in advance.

In the real world, capturing RGB images is more convenient than reconstructing a 3-D model. Moreover, as the visual perception is more and more applied to the robot’s grasping operation task [18,19,20], many works try to extract the target features that best reflect the grasping characteristics from the visual information to guide the robot complete the grasping operation of the target. Saxena et al. [18] extracted artificially designed image features from the training data set, trained a grasping probability model, completed the task of learning the target grasping point from the picture, and successfully applied it to the actual field. Add the deep image features, Le et al. [19] realized the learning of multi-touch point grasping of the target object. The algorithm clarified the contact point between each finger and the target object, which can be extended to different multi-finger dexterous hand grasping operations.

Recent years, deep learning has provided the possibility of detecting grasping directly from RGB or RGB-D images with its powerful feature extraction and generalization capabilities [20]. Lenz et al. [2] used two neural networks in series to detect the grasping position in the RGB-D image. The first network has a small scale and is responsible for eliminating candidate grasping positions with a low probability. The second network has a larger scale and can extract more features, judge the remaining candidate positions, and obtain the optimal grasping position. Redmon et al. [3] proposed a real-time and accurate grasp detection algorithm based on convolutional neural network for RGB-D image data, which can simultaneously realize the classification of the object to be grasped and the regression of the grasping position. The algorithm can reach a processing speed of 13 frames per second on the GPU. Kurma et al. [4] used the residual network as the feature extraction layer, and trained the Uni-modal model using only RGB data and the Multi-modal model using RGB-D data. Multi-modal uses two residual networks to extract color features and depth features at the same time and achieves better detection results. Guo [5] et al. fused the visual and tactile information in the grasping process, and propose a new hybrid neural network to process multi-modal information and detect the optimal grasping position. The author established a THU grasping dataset for model training and achieved good results. Chu et al. [11] based on the RPN network (Region Proposals Network) proposed a model that can be used to simultaneously determine the grasping position of multiple targets in an RGB-D image and achieved good results. Depierre et al. [21] proposed a neural network with a scorer which evaluates the graspability of a given position and introduce a novel loss function which correlates regression of grasp parameters with graspability score. Based on the Event-Stream dataset, Li et al. [22] develop a deep neural network for grasping detection which consider the angle learning problem as classification instead of regression. This work provides a large-scale and well-annotated dataset, and promotes the neuromorphic vision applications in robot grasp. Zhou et al. [6] proposed the oriented anchor box mechanism that assigns the reference rectangles with different default rotation angles, which achieved the current best results on the Cornell dataset. The above are all single-object scenes for the Cornell dataset, and some works involve grasping in scenes with cluttered objects. Guo et al. [23] trained a deep network on a fruit dataset containing 352 RGB images to simultaneously detect the most exposed object and its best grasp. However, their model can only output the grasp belonging to the most exposed object, without the perception and understanding of the overall environment, which limits the use of algorithms. The algorithms proposed in [7,8,9,10,11] only focus on the detection of grasps in densely and cluttered scenes, rather than what the grasped objects are. Vohra et al. [24] proposed a method to estimates the object contour in the point cloud and predicts the grasp pose along with the object skeleton in the image plane. Zhang et al. [13] proposed a robot grasping detection algorithm ROI-GD based on a region of interest (ROI), which can detect objects and their corresponding grasps at the same time and has better performance in multi-object scenes.

Nowadays, methods based on deep learning have dominated, most of which are anchor-based ideas and list a large number of candidate grasps. However, for humans, to grasp an object, first determine the grasp point of the object instead of selecting a large number of candidate grasps. Therefore, we propose a grasp detection algorithm based on key-points.

## 3. The Proposed Method

### 3.1. Problem Formulation

**Figure 2:**
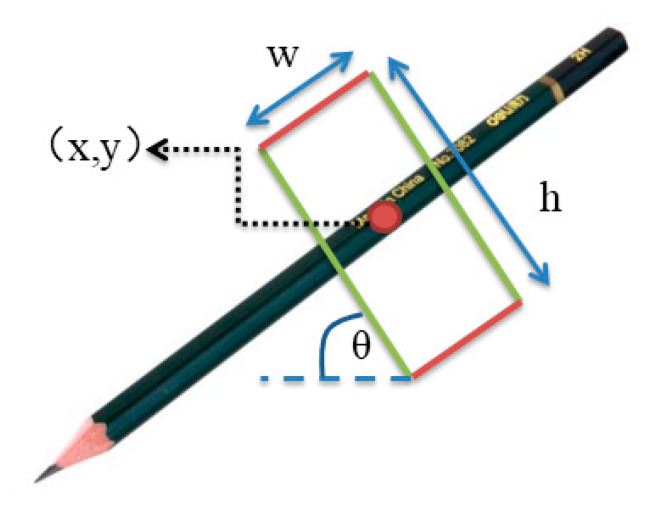
For a pencil, showing its grasp center at (x,y) oriented by an angle of θ from its horizontal axis. The rectangle has a width and height of w and h respectively.

For single-object scenes, the five-dimensional robot grasping representation proposed by Lenz et al. [2] is widely used for grasping detection with parallel graspers. We use this method in a single-object scene, and the five-dimensional grasp configuration *g* is represented as follow:

(1) g=(x,y,w,h,θ)

where (x,y) corresponds to the center of grasp rectangle, h is the height of parallel plates, w is the maximum distance between parallel plates and θ is the orientation of grasp rectangle with respect to the horizontal axis [4]. As shown in Figure 2, an example has been represented.

To complete the task of grasping a specified object in multi-object scenes, we also need to know the object to which the grasp belongs. We extended the five-dimensional robotic grasp representation to including object class as follows:

(2) $$g=(x_{gd},y_{gd},\theta_{gd},w_{gd},h_{gd},cls_{gd})$$

where $cls_{gd}$ represents the object to which the grasp belongs.

### 3.2. Network Architecture

**Figure 3:**
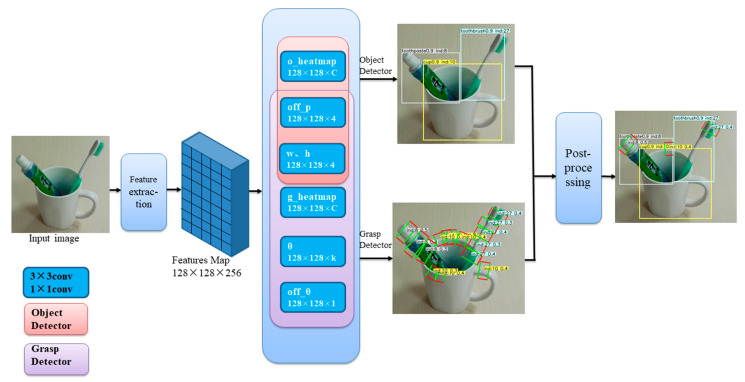
The overall structure of the network. The dark blue boxes represent 3 × 3 and 1 × 1 convolutions, which are used to generate outputs in each dimension; light red represents the object detector part, and light purple represents the grasp detector part.

Our scheme consists of a feature extractor, object detector, grasps detector and other related attributes, as shown in Figure 3. Because the correctness of key points plays a decisive role in the detection results, here we use several different fully convolutional encoder-decoder networks as feature extractors. We tried stacked hourglass networks [25,26] and deep layer aggregation (DLA) [27] in our experiments. Although the hourglass network is powerful, the network is quite large and slow, comprehensive speed and accuracy, choose DLA-34.

Our scheme is one-stage detector, which directly predict grasps and objects from the feature maps generated by feature extractor. Let $I\in R^{W\times H\times 3}$ be an input image of width *W* and height *H*. Our aim is to produce two keypoint heatmaps ${\hat{H}}_{o\left(g\right)}\in {\left[0,1\right]}^{\frac{W}{S}\times \frac{H}{S}\times C}$ one prediction grasp and one prediction object, where *S* is the output stride to downsample the output prediction (We use the default output stride of *S* = 4 in literature [26,28,29,30,31,32]) and *C* is the number of object categories. A prediction ${\hat{H}}_{o\left(g\right)}\_xyc=1$ corresponds to a detected keypoint, while ${\hat{H}}_{o\left(g\right)}\_xyc=0$ is background. Then regress to other attributes, for example, $w,h\in \frac{W}{S}\times \frac{H}{S}\times 4$ contains the size of the object and the grasp; we use a classification method for angles, so *k* in $\theta \in \frac{W}{S}\times \frac{H}{S}\times k$ represents the number of angle categories. if *k* is equal to 6, the default rotation angles are 75°, 45°, 15°, −15°, −45° and −75°. In addition, we also corrected the offset caused by downsampling through $off\_p\in \frac{W}{S}\times \frac{H}{S}\times 4$, and fine-tuned the classified angle through $off\_\theta \in \frac{W}{S}\times \frac{H}{S}\times 1$.

### 3.3. Keypoint Estimate Mechanism for Training

We model an object or a grasp as a single point—the center point of its bounding box. The detector uses keypoint estimation to find the center point and regress to all other object attributes such as size, direction, etc. We predict two sets of heatmaps, one set for object and the other for grasp. Each heatmaps has *C* channels, where *C* is the number of categories, and is of size $\frac{H}{S}\times \frac{W}{S}$. They have no background channel. Each channel is a binary mask representing the center point of the object or grasp for a class. In the network, the key-point estimation is done by Gaussian function. First, for each center point $p\in R^{2}$ of the object or grasp, we calculate an equivalent point $\tilde{p}\in R^{2}$ after downsampling. Then calculate an object size-adaptive standard deviation $\;\sigma_{p}$ determined by the object size for each point [25]. Finally, the center point of the object and grasp is scattered on the key-point heatmap ${\hat{H}}_{o\left(g\right)}\in {\left[0,1\right]}^{\frac{W}{S}\times \frac{H}{S}\times C}$ by Gaussian kernel function. If two Gaussians of the same class overlap, we take the element-wise maximum [4]. The loss function of the training keypoint heatmap uses the penalty-reduced pixel-wise logistic regression with focal loss in [32]:

(3) $$L_{h}=-\frac{1}{N}\sum_{C=1}^{C}\sum_{i=1}^{\frac{H}{R}}\sum_{j=1}^{\frac{W}{R}}\{{}_{{\left(1-H_{cij}\right)}^{\beta }{\left(\overset{\wedge }{H_{cij}}\right)}^{\alpha }\log{\left(1-\overset{\wedge }{H_{cij}}\right)}_{}\;otherwise}^{{\left(1-\overset{\wedge }{H_{cij}}\right)}^{\alpha }\log{\left(\overset{\wedge }{H_{cij}}\right)}_{}\;if\;\overset{}{H_{cij}=1}}$$

(4) $$L_{h}=-\frac{1}{N}\sum_{c=1}^{C}\sum_{x=1}^{\frac{H}{S}}\sum_{y=1}^{\frac{W}{S}}\{{}_{{\left(1-H_{xyc}\right)}^{\beta }{\left(\overset{\wedge }{H_{xyc}}\right)}^{\alpha }\log{\left(1-\overset{\wedge }{H_{xyc}}\right)}_{}{}_{}\;otherwise}^{{\left(1-\overset{\wedge }{H_{xyc}}\right)}^{\alpha }\log{\left(\overset{\wedge }{H_{xyc}}\right)}_{}\;if\;\overset{}{H_{xyc}=1}}$$

where *α* and *β* are hyper-parameters of the focal loss [32], and *N* is the number of keypoints of the objects or grasps in image. The normalization by *N* is chosen as to normalize all positive focal loss instances to 1. We use *α* = 2 and *β* = 4 in all our experiments, following Law and Deng [25]. With the Gaussian bumps encoded in $H_{xyc}$, the $\left(1-H_{xyc}\right)$ term reduces the penalty around the ground truth locations.

### 3.4. Loss Function

Since we predicted two heatmaps of the objects and grasps, $L_{h}$ contains two parts: $L_{h\_o}$ used to predict the center of the objects and $L_{h\_g}$ used to predict the center of the grasps.

(5) $$L_{h}=L_{h\_o}+L_{h\_g}$$

(6) $$L_{h\_o}=-\frac{1}{N_{o}}\sum_{c=1}^{C}\sum_{x=1}^{\frac{H}{S}}\sum_{y=1}^{\frac{W}{S}}\{{}_{{\left(1-H_{o\_xyc}\right)}^{\beta }{\left(\overset{\wedge }{H_{o\_xyc}}\right)}^{\alpha }\log{\left(1-\overset{\wedge }{H_{o\_xyc}}\right)}_{}{}_{}\;otherwise}^{{\left(1-\overset{\wedge }{H_{o\_xyc}}\right)}^{\alpha }\log{\left(\overset{\wedge }{H_{o\_xyc}}\right)}_{}\;if\;\overset{}{H_{o\_xyc}=1}}$$

(7) $$L_{h\_g}=-\frac{1}{N_{g}}\sum_{c=1}^{C}\sum_{x=1}^{\frac{H}{S}}\sum_{y=1}^{\frac{W}{S}}\{{}_{{\left(1-H_{g\_xyc}\right)}^{\beta }{\left(\overset{\wedge }{H_{g\_xyc}}\right)}^{\alpha }\log{\left(1-\overset{\wedge }{H_{g\_xyc}}\right)}_{}{}_{}\;otherwise}^{{\left(1-\overset{\wedge }{H_{g\_xyc}}\right)}^{\alpha }\log{\left(\overset{\wedge }{H_{g\_xyc}}\right)}_{}{}_{}\;if_{}\;\overset{}{H_{g\_xyc}=1}}$$

When predicting keypoints, we downsampled the center point and rounded down the coordinates of the points after downsampling. That is, a position (x,y) in the image is mapped to position $\left(\frac{x}{S},\frac{y}{S}\right)\;$ in the heatmaps where *S* is the downsampling factor. In this way, when mapping from the heatmaps back to the input image, errors will occur, which will have a greater impact on small objects. For this reason, we predict the keypoint position offset $off\_p\in \frac{W}{S}\times \frac{H}{S}\times 4$ to correct the position of the center point.

(8) $$off{\_}_{p_{k}}=\left(\frac{x_{k}}{S}-[\frac{x_{k}}{S}],\frac{y_{k}}{S}-[\frac{y_{k}}{S}]\right)$$

where $off\_p_{k}$ is the offset of the objects center and grasps center, $x_{k}$ and $y_{k}$ are the *x* and *y* coordinate in the image for center k.

We adopt *L*1 loss for regression loss.

(9) $$L_{off\_p}=-\frac{1}{N_{o}}\sum_{k=1}^{N_{o}}\left|{\overset{\wedge }{off\_p}}_{k}-{\overset{}{off\_p}}_{k}\right|-\frac{1}{N_{g}}\sum_{k=1}^{N_{g}}\left|{\overset{\wedge }{off\_p}}_{k}-{\overset{}{off\_p}}_{k}\right|$$

where *N_o_* and *N_g_* are the number of objects and grasps on a picture respectively. Since we use the classification method to predict the angle *θ*, here we use cross entropy for training:

(10) $$L_{\theta }=-\sum_{k=1}^{N_{g}}\log(p_{\theta }^{\left(k\right)})$$

In order to improve the accuracy of the prediction, we also predict the angle offset $off\_\theta_{k}=\left({\hat{\theta }}_{k}-\theta_{k}\right)$ and adopt *L*1 loss for regression loss.

(11) $$L_{off\_\theta }=-\frac{1}{N_{g}}\sum_{k=1}^{N_{g}}\left|{\overset{\wedge }{off\_\theta }}_{k}-{\overset{}{off\_\theta }}_{k}\right|$$

For the size $s_{k}=\left(w^{k},h^{k}\right)$ of the objects and the grasps, we use a single size prediction $\hat{S}\in R^{\frac{W}{S}\times \frac{H}{S}\times 2}$ to predict it and adopt *L*1 loss for regression loss.

(12) $$L_{w\_h}=-\frac{1}{N_{o}}\sum_{k=1}^{N_{o}}\left|\overset{\wedge }{s_{k}}-\overset{}{s_{k}}\right|-\frac{1}{N_{g}}\sum_{k=1}^{N_{g}}\left|\overset{\wedge }{s_{k}}-\overset{}{s_{k}}\right|$$

Finally, our loss function is defined as:

(13) $$L=L_{h}+\lambda_{w\_h}L_{w\_h}+\lambda_{\theta }L_{\theta }+\lambda_{off\_p}L_{off\_p}+\lambda_{{}_{off\_\theta }}L_{off\_\theta }$$

We set $\lambda_{w\_h}=0.1$, $\lambda_{off\_p}=1$ and $\lambda_{off\_\theta }=1$ in all our experiments. In addition, the angle is an important factor for grasps, so $\lambda_{\theta }=1$. Moreover, we use a single network to predict the keypoint ${\hat{H}}_{o\left(g\right)}$, offset ${\overset{\wedge }{off\_p}}_{k}$, angle $\;\hat{\theta }$ and size ${\hat{S}}_{k}$. The network predicts a total of 2C+(7+k) outputs at each location, where *k* is the number of angle categories.

### 3.5. Matching Strategy

The purpose of post-processing is how to get the grasps of each object, so a good matching strategy is obviously very important. Firstly, non-maximum suppression (NMS) is performed on the detected objects and grasps to eliminate unnecessary detection results; then the objects and grasps are grouped according to the category information of the objects and grasps; then based on the following formula, remove the grasps that are less than the threshold.

(14) $$IOU=\frac{A_{od}\cap A_{gd}}{A_{od}\cup A_{gd}}$$

where $A_{od}$ is the area of the object detection rectangle and $A_{gd}$ is the area of the grasp detection rectangle. $A_{od}\cup A_{gd}$ is union of these two rectangles.${\;A}_{\mathrm{od}}\cap A_{\mathrm{gd}}$ is the intersection of these two rectangles. Finally, among the grasp candidates whose *IOU* exceeds a certain threshold (we set *IOU* = 0.4), the grasp closest to the target center is selected as the grasp configuration.

## 4. Experiments

### 4.1. Dataset

The algorithm proposed in this paper is faced with complex multi-object scenes. We use the VMRD dataset [14] to verify the performance of the algorithm. Because a large number of previous grasp detection algorithms are for single-object scenes, it is necessary to verify the effect of the proposed algorithm in single-object scenes. Here we use Cornell dataset [2] for verification.

**Figure 4:**
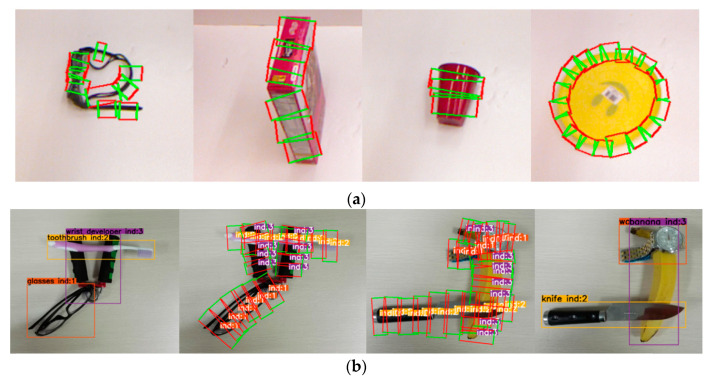
Dataset example: (**a**) Examples of Cornell Dataset; (**b**) Examples of VMRD. The two sides are the object detection results and the middle is the corresponding grasp detection results.

The Cornell dataset [2] consists of 855 images (RGB-D) of 240 different objects, and the ground truth tags are some graspable/non-graspable rectangles, as shown in Figure 4a. We divided the training set and the test set into four to one and adopted the object-wise splitting and the image-wise splitting criteria used in previous works [2] for performance verification.

1. Image-wise split splits all the images of the dataset randomly into training set and validation set. This is to test the generalization ability of the network to detect new objects.
2. Object-wise split splits the object instances randomly into train and validation set. The training and validation data sets do not have instances in the other’s dataset. That is, all pictures of the same instance are assigned to the same dataset. This is to test the generalization ability of the network to new instances.

VMRD consists of 4233 train data and 450 test data (RGB images) as shown in Figure 4b. This dataset contains 31 object categories and there are 2 to 5 stacked objects in each image. Each object includes category information, bounding box location, the index of the current object and indexes of its parent nodes and child nodes. Moreover, each grasp includes the index of the object it belongs to, bounding box location. The affiliation between the objects and the grasps is determined by the index. For example, as shown in the left image of Figure 4b, the index of the glasses is 1, so all the grasps with index 1 in the second image belong to the glasses.

Deep neural networks require a large amount of labeled data for training. However, data is usually not easily available and labeling is usually expensive. The problem is usually solved from two aspects. First, perform pre-training on a larger dataset. Second, expand the dataset through data augmentation. In this paper, backbone of network are initialized with ImageNet [27] pretrain. We use random flip, random scaling cropping, and color jittering as data augmentation.

### 4.2. Implementation Details

Our network is implemented on PyTorch. GPUs used to train the network are all Tesla T4 with 16 GB memory. We train with a batch-size of 32 (on 2 GPUs) and learning rate 5 × 10^−4^ for 70 epochs, with learning rate dropped 10× at 30 and 60 epochs, respectively. We use Adam to optimize our schemes. For each of the scheme, we adopt the same training regimen. The input resolution of the network is 512 × 512 and the feature map of 128 × 128 resolution is generated after feature extraction. Then fit the keypoints and regress to other attributes based on the feature map.

After detecting the grasps from the RGB image, it is necessary to combine the depth information to obtain the grasp point and the approaching vector. First find the coordinates $\left(x_{minD},y_{minD}\right)$ of the smallest depth point within 5 pixels near the center point (x,y) of the grasp box; then get a square area D with a side length of 10 pixels near point $\left(x_{minD},y_{minD}\right)$ from the depth map; then use the camera internal parameters to convert all the 2D pixel coordinates in square area D into 3D coordinate points in the camera coordinate system to obtain a point cloud P. Among them $\left(x_{minD},y_{minD}\right)$ converted 3D points are the final grasp points. Then calculate the surface normal vector of the point cloud P, and find the average n of all surface normal vectors. The approach vector is −n.

### 4.3. Metrics

For single-object scenes, we use the “rectangle-metric” proposed in [1] to evaluate the network’s grasp detection ability. In this metric, a grasp that meets the following two conditions is considered a good grasp:

1. the difference between the predicted grasp rotation angle and the ground-truth grasp rotation angle is less than 30°.
2. the Jaccard index of the predicted grasp and the ground-truth is more than 25%. The Jaccard index for a predicted rectangle g and a ground-truth rectangle $\hat{g}$ defined as:
  (15) $$J(g,\overset{\wedge }{g})=\frac{A_{g}\cap A_{\overset{\wedge }{g}}}{A_{g}\cup A_{\overset{\wedge }{g}}}$$
  where $A_{g}$ is the area of predicted grasp rectangle and $A_{\hat{g}}$ is the area of ground-truth grasp rectangle. $A_{g}\cup A_{\hat{g}}$ is union of these two rectangles.$\;A_{g}\cap A_{\hat{g}}$ is the intersection of these two rectangles.

To compare with the previous algorithm, we use the (*o*, *g*) metric proposed by [13] to evaluate the algorithm in the multi-object scenes. A detection (*o*, *g*) is considered a True Positive when it meets the following conditions:

1. A detection (*o*, *g*) includes object detection result *o* = (*Bo*, *Co*) and top-1 grasp detection result g, where Bo is the position of the object predicted by the network and *Co* is the predicted category.
2. The object *o* is detected correctly, which means that the predicted category *Co* is the same as ground-truth and the IOU between the predicted position *Bo* and ground-truth is greater than 0.5.
3. The Top-1 grasp *g* is detected correctly, which means that the predicted Top-1 grasp has a rotation angle difference less than 30° and Jaccard Index more than 0.25 with at least one ground truth grasp rectangle belonging to the object.

## 5. Results

### 5.1. Validation Results on Cornell Dataset

**Figure 5:**
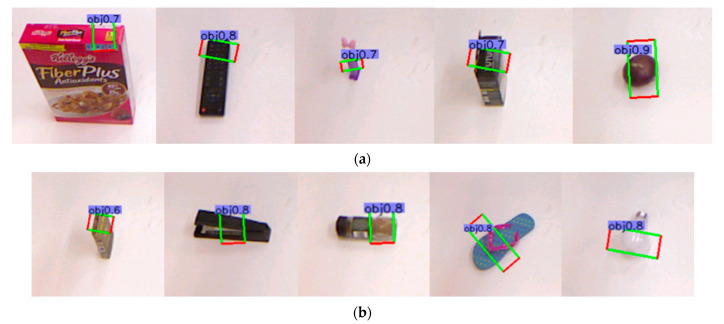
Prediction result: (**a**) Correct prediction result on Cornell Grasp Dataset; (**b**) Failures prediction result on Cornell Grasp Dataset.

In order to verify the algorithm proposed in this paper, first use the Cornell dataset to verify the single-object scene. As shown in Table 1, the accuracy of previous work on the Cornell dataset is list. Moreover, the verification results of this work on the Cornell dataset are shown in Table 1 and Figure 5a. We achieved an accuracy of 96.05% and 96.5% in image-wise and object-wise split. Compared with state-of-the-art algorithm [6], it is competitive. Some error detections of the dataset are shown in Figure 5b. It can be seen from the figure that these error detections are feasible for robot grasping. In other words, the labeling of the dataset is not exhaustive. This also further illustrates the rationality of our classification method for angle prediction.

### 5.2. Validation Results on VMRD Dataset

For multi-object scenes, we use the VMRD dataset for verification. As shown in Table 2, the results of our ablation experiments on the VMRD dataset are list. It can be seen from the optimization process that the number of angle categories *k* has a greater impact on the detection result. The value of *k* is negatively correlated with the angle range corresponding to each angle category. The larger the angle range, the worse the prediction accuracy, and the smaller the angle range, the better the prediction accuracy, but the difficulty of learning increases. The comprehensive experimental result *k* is the best. In addition, Focal Loss dynamically adjusts the Cross Entropy according to the confidence. When the confidence of the correct prediction increases, the weight coefficient of loss will gradually decay to 0, so that the loss function of model training pays more attention to the hard cases, and a large number of easy examples contribute very little to it. So, we use Focal Loss to improve the detection results.

**Figure 6:**
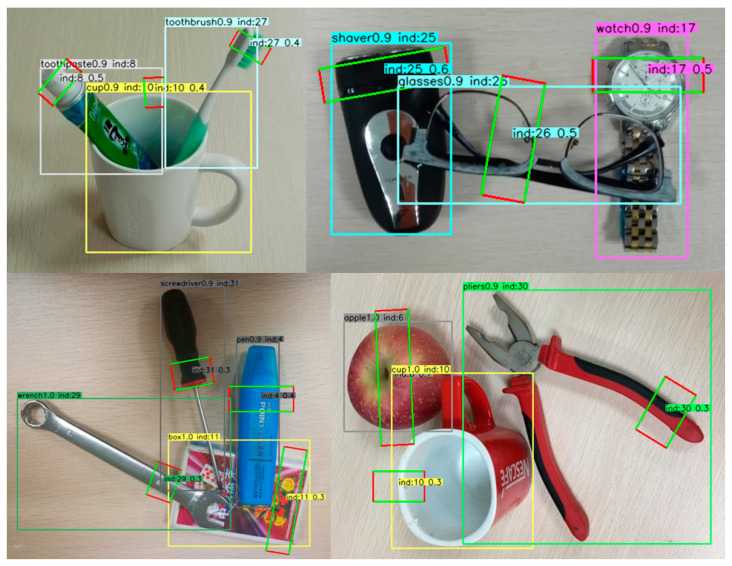
Examples of prediction result on VMRD.

As shown in Table 3, accuracy of the previous works and our proposed method on VMRD Dataset are listed. Our proposed method yielded state-of-the-art performance of 74.3% mAPg. And as shown in Figure 6, the verification result is listed.

### 5.3. Robot Experiment

In our robot experiment, we use a UR5 robot with six degrees of freedom as the executor. The UR5 robot can handle automated tasks up to 5 kg and has an extended radius of 850 mm, which is perfect for lightweight collaborative processes. The grasping plane and the robot base are on the same plane. The camera uses RealSense depth camera, which is installed with eyes on hands. In the process of perception and inference, we use RGB images, and add depth information to the calibration process to calculate the grasp points and approaching vector during the grasping process.

**Figure 7:**
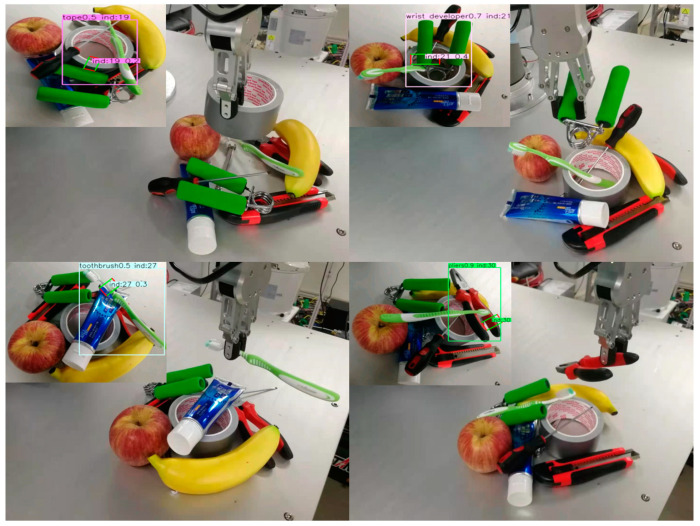
Robot experiment. The top-left corner of the picture is the detection result, and the bottom-right corner is the corresponding grasping operation.

In this paper, the problem we want to solve is to grasp a specific object in a multi-object scene. In order to test the performance of the network in the real world, we select several objects and place them on the grasping plane chaotically to generate various multi-object scenes. For each scene, we recognize and grasp the specified object. We try to grasp each of objects 10 times and record the number of successes. There are ten kinds of objects specifically used in the experiment, namely apples, bananas, wrist developer, tape, toothpaste, wrenches, pliers, screwdrivers, knife and toothbrush. The experimental results are shown in Table 4 and Figure 7. Accuracy of the previous works and our proposed method on robot grasping experiment are shown in Table 5. In single-object scenes, the overall success rates of our algorithm in prediction and execution are 98% and 94%, respectively. In multi-object scenes, the overall success rates of our algorithm in prediction and execution are 94% and 87%, respectively.

## 6. Discussion

The limitation of this scheme is: in our algorithm, the specified object to be grasped must meet the condition of not being covered by other objects. When the specified object is below another object, our algorithm can detect the grasps but cannot perform the grasping operation successfully because direct grasping will damage the above object. In this case, if we know the operational relationship between the objects, we can move the objects above the specified object in order, and then grasping the specified object. This avoids damage to the above objects. Therefore, it is important to know the operational relationship between objects.

## 7. Conclusions and Future Work

We propose a keypoint-based grasp detection algorithm, which models the objects and grasps as a point and regresses to all other object attributes, such as size, direction, etc. Experimental results demonstrate that the accuracy of this method is 74.3% and 96.05% in the multi-object grasp dataset VMRD and single-object grasp dataset Cornell dataset, respectively. Through the analysis of the failure prediction result of the Cornell dataset, we found that these failure detection results are still a good grasp because the dataset annotation is not exhaustive. The results show that this algorithm is specifically competitive with the state-of-the-art grasp detection algorithm in single-object scenes, and it performs better in multi-objects scenes. Robot experiments demonstrate that this method can help robots grasp the target in single-object and multi-object scenes with success rates of 94% and 87%, respectively.

In future work, the design of the representation form of the operational relationship between overlapping objects and how to efficiently detect the operational relationship between objects are our research focus. These are the keys to achieve grasps in overlapping scenes.

## Figures and Tables

**Table 1:** Performance of different algorithm on Cornell Grasp Dataset.

Approach	Algorithm	Accuracy (%)	
		Image-Wise	Object-Wise
Jiang et al. [1]	Fast Search	60.5	58.3
Lenz et al. [2]	SAE, struct. reg. Two stage	73.9	75.6
Redmon et al. [3]	AlexNet, MultiGrasp	88.0	87.1
Kumra et al. [4]	ResNet-50 × 2, Multi-model Grasp Predictor	89.21	88.96
Guo et al. [5]	ZF-net, Hybrid network, 3 scales and 3 aspect ratios	93.2	89.1
Chu et al. [11]	VGG-16 model	95.5	91.7
	ResNet-50 model	96.0	96.1
Zhou et al. [6]	ResNet-50 FCGN	97.7	94.9
	ResNet-101 FCGN	97.7	96.6
the proposed scheme	keypoint-based scheme	96.05	96.5

**Table 2:** Ablation experiment results on VMRD.

k ^1^	Loss	mAPg (%) ^2^
6	Cross Entropy	57.3
9	Cross Entropy	64.2
12	Cross Entropy	69.3
18	Cross Entropy	72.6
18	Focal Loss	74.3

^1^ k: Number of angle categories. ^2^ mAPg: mAP with grasp.

**Table 3:** Performance of different algorithm on VMRD.

Approach	Algorithm	mAPg (%)
Zhou et al. [13]	Faster-RCNN [31] + FCGN [6]	54.5
Zhou et al. [13]	ROI-GD	68.2
the proposed scheme	keypoint-based scheme	74.3

**Table 4:** Robotic grasp experiment results.

Object	Single-Object Scenes		Multi-Object Scenes	
	Prediction	Grasping	Prediction	Grasping
Knife	8/10	8/10	7/10	7/10
Bananas	10/10	10/10	10/10	10/10
Toothbrush	10/10	9/10	10/10	8/10
Toothpaste	10/10	10/10	10/10	10/10
Wrenches	10/10	10/10	10/10	9/10
Wrist developer	10/10	10/10	8/10	7/10
Screwdrivers	10/10	8/10	10/10	10/10
Apples	10/10	10/10	10/10	9/10
Pliers	10/10	9/10	9/10	7/10
Tape	10/10	10/10	10/10	10/10
Accuracy (%)	98	94	94	87

**Table 5:** Accuracy of different algorithm on robot grasping experiment.

Approach	Algorithm	Accuracy (%)	
		Single-Object Scenes	Multi-Object Scenes
Zhou et al. [13]	ROI-GD	92.5	83.75
the proposed scheme	keypoint-based scheme	94	87
